# How Artificial Intelligence Will Change the Future of Tourism Industry: The Practice in China

**DOI:** 10.1007/978-3-030-65785-7_7

**Published:** 2020-11-28

**Authors:** Yanzheng Tuo, Lanyu Ning, Aiyuan Zhu

**Affiliations:** 1 Department for Informatics, Technical University of Munich, Garching bei München, Bayern Germany; 2 Smart Tourism Education Platform (STEP) College of Hotel and Tourism Management, Kyung Hee University, Seoul, Korea (Republic of); 3 Department of Tourism and Service Management, MODUL University Vienna, Vienna, Wien Austria; 4 Nankai University, Tianjin, China; 5 College of Tourism and Service Management, Nankai University, Tianjin, China

**Keywords:** Artificial intelligence, Tourism industry, Robots, Tourist experience, Privacy

## Abstract

In the future, artificial intelligence (AI) is likely to substantially change both the tourism industry and tourist behavior. At present, research on artificial intelligence and tourism is receiving widespread attention, but most of them focus on a certain subject or a specific aspect of the tourism industry. For example, artificial intelligence influences the behavior of tourists and tourism enterprises. The analysis of the impact of artificial intelligence on the tourism industry as a system is still insufficient. Therefore, this research proposes a multi-dimensional framework from an industry perspective based on the existing definition of artificial intelligence. The framework involves three aspects: the level of intelligence, task types, and whether artificial intelligence is embedded in robots. The authors use a large number of Chinese practice cases to investigate how AI affects the tourism industry, then put forward a research agenda to analyze how destination government, tourism enterprises and tourist experience will change in the future. Finally, they highlight important issues related to privacy, prejudice and ethics.

## Introduction

As an interdisciplinary, super-level original technique, artificial intelligence is profoundly changing the human society and global landscape [1, 2]. The subversive nature of artificial intelligence technology is reflected in the following aspects: (1) It replaces and performs better than manpower in highly repetitive and labor-intensive industries [3, 4]^.^(2) It can provide interactive experience and deep involvement. (3) Scene design based on the artificial intelligence can provide personalized and customized products and services for any industry [5].

Artificial intelligence plays a key role in a series of innovations and applications in the tourism industry, and is deeply changing the experience of tourism and service [6]. The global spread of COVID-19 at the beginning of 2020 has caused worldwide economic stagnation. Thanks to the artificial intelligence, the wide application of Telemedicine, UAV monitoring, robot nursing in hospitals, Google Duplex, Siri, customer service speech has not only replaced medical staff to complete many high-risk tasks, but also become a human “special companion” in extraordinary times.

In real life, artificial intelligence has been widely used in tourism and service industry. However, in the academic field, most of the existing research is concentrated on the field of information science, exploring the artificial intelligence technology itself [7] and the ethical issues brought by the technology [8], lack of research based on an interdisciplinary and macro perspective. Scholars mainly study the impact of artificial intelligence technology on employment [9], individuals’ anxiety, panic and other negative emotions caused by the substitution effect of artificial intelligence technology on humans [10]. In addition, there have been studies on the effect of AI on the tourism and service industry, marketing [11], strategy [12], and retail [13]. Most of them are speculative studies lacking scientific evidence. Above questions can be summarized into three aspects: (1) Existing studies have not analyzed the connotation and characteristics of the impact of technology on the tourism industry based on the uniqueness of artificial intelligence technology. (2) Since artificial intelligence is an emerging field, research on the mechanism by which artificial intelligence affects the tourism industry is very scarce, which makes it impossible to answer the impact of artificial intelligence on the tourism industry from the internal mechanism. (3) There are few existing studies on the side effects of artificial intelligence on the tourism industry, which leads to relevant policies, corporate management, and public management that cannot recognize their particularity.

Existing studies rarely systematically explain how artificial intelligence will affect the tourism industry and how artificial intelligence will develop in the future. Out of practical and theoretical demands, this article attempted to put forward a research framework on how AI influences and changes the tourism industry. Based on insights from marketing (more broadly, business), social sciences (e.g. psychology, sociology) and computer science/robotics, we proposed a framework which can help tourists, tourism enterprises, governments and destinations foresee how artificial intelligence will develop and evolve.

## Literature Review

### Artificial Intelligence

Most definitions of artificial intelligence focus on it as a subfield of computer science or in terms of how machines can mimichuman intelligence [14]^.^ Since the emergence of artificial intelligence technology is inseparable from the rapid development of computer technology, some scholars tend to define it from the perspective of computer science, believing that it is “the scientific study of the computational principles behind the ideology and intelligent behavior” [15]. Others are concerned about the relationship between artificial intelligence and human intelligence. They divide AI into the following three types: Weak AI that can replace humans in some aspects, Strong AI with a high degree of perception of the outside world and automatic learning capabilities, and Artificial Super Intelligence, which has super artificial intelligence that far exceeds human intelligence [16].

Another way to describe artificial intelligence does not depend on its underlying technology, but on its marketing and commercial applications [17]. For consumers, AI gives customized buyer experience. For producers and retailers, AI allows them to gain sharper predicting tools that ensure the making of sharper business decisions [18]. This research is mainly based on the latter definition and discusses its impact on the tourism industry.

### Tourism Industry

Some researchers try to use the knowledge of network science to understand it. They believe that tourism is a dynamic and complex system [19]. A central point is that complex systems are investigated as holistic entities, given the impossibility to comprehend all their manifestations as compositions of individual traits and behaviors [20].

Gunn [21] first proposed the concept of the functioning tourism system, emphasizing that it consists of two parts: demand and supply. Some researchers pay more attention to the entire tourism industry from the supply side, that is, to focus on the competitiveness of tourism destinations at a more micro level. They believe that the destination can be described as a complex networked system, where local actors (public and private) and organizations are the nodes, and the relationships between them are the links [21].

In this article, the research object is regarded as a tourism system composed of dynamic and interdependent tourism elements, including governments of tourism destinations, tourism enterprises, and tourists.

### Artificial Intelligence, Tourism and Tourism Industry

From the perspective of tourism enterprises as the supply side, artificial intelligence is in the stage of growth from Weak AI to Strong AI, and its impact on employment is continuously increasing [4]. The service process is adjusted, and the cost and method of management are also affected. As a result, the most obvious financial benefit is labour costs savings [22].

From the perspective of tourists, artificial intelligence has a great impact on consumers and their experience. In the Web of science database, searching with Artificial Intelligence and Consume/Consumption/Consumer as the subject keywords, a total of 5216 related documents were retrieved, of which the number of studies from 2011 to 2014 was relatively small. Since 2015, there has been an upward trend in the number of studies focused on computer science, communications science, and engineering, but there has been less research in the fields of social science, economics, and management science. At present, smart customer service, accurate information push, robot sensing services and other methods have had a great impact on consumer needs, preferences, decision-making and experience [23]. For example, it has been found that consumers who find that they are talking to robots will think that robots have insufficient expertise and believe that their empathy is worse [24]. In the context of business change, artificial intelligence also has a certain impact on customer experience. Artificial intelligence can provide customers with customized and personalized services; realize scene experience; meet emotional needs; better meet basic needs. The application of artificial intelligence technology in the retail field can effectively promote consumer interaction and pleasure in scene marketing, improve customer shopping satisfaction and customer loyalty, enhance consumer desire and expand consumption [25].

## Research Design

Based on the insights of marketing, social science and computer science/robotics, Davenport T et al. [26] proposed a research framework system to comprehensively predict the impact of artificial intelligence on marketing. They divided the system into three dimensions: the level of intelligence, task types, and whether the AI is embedded in a robot. The level of intelligence includes task automation and context awareness. The former refers to the application of artificial intelligence which can perform standardized and consistent tasks. The latter encompasses different forms of intelligence that require computation and algorithms to “learn how to learn”. Task types refer to whether an AI program analyzes numbers or non-numeric data. Analyzing numbers is easier than other forms, but in fact, the majority of data is non-digital, which needs more powerful intelligent analysis capabilities. As for the forms of artificial intelligence, one is virtual, such as some digital platforms used on mobile phones; the other is based on entities. This multi-dimensional thinking or data-driven method enables us to have a deeper and comprehensive understanding of the nature and internal development laws of things. Therefore, this paper uses Davenport T’s research framework to conduct cross-over research in a comprehensive and systematic way.

This research is aimed to help destination management organization and tourism companies predict how artificial intelligence may develop. We considered three perspectives: governments, tourism enterprises, and tourists. In each perspective, there are three dimensions related to artificial intelligence: the level of intelligence, task types, and forms of AI. We extracted topics and questions from real cases, and integrated them to form the framework of the impact of AI on governments, tourism enterprises, tourists and future issues.

**Table 1.** Research design

The main body of tourism	Forms of artificial intelligence	
The government (Destination Management Organization)	Level of intelligence	Task automation
		Context awareness
	Task types	Numbers
		Non-numeric data
	Forms of AI	Digital form
		Robot form
Tourism enterprises	Level of intelligence	Task automation
		Context awareness
	Task types	Numbers
		Non-numeric data
	Forms of AI	Digital form
		Robot form
Tourist	Level of intelligence	Task automation
		Context awareness
	Task types	Numbers
		Non-numeric data
	Forms of AI	Digital form
		Robot form

## Findings

### The Impact of Artificial Intelligence on Tourism Destination Governments

#### Level of Intelligence.

*Task automation.* In China, the instrumental value of artificial intelligence and “intelligence” as an important direction of social governance in destinations have been highly valued by governments at all levels [27]. A series of intelligent service management platforms represented by Hangzhou “City Brain” hope to give full play to government management functions with the support of AI. The “City Brain” can automatically sense and process real-time emergencies in various public services at tourist destinations. The traffic system of the “City Brain” scans the traffic conditions of the city’s roads every 2 min and automatically warns in advance of possible changes in road conditions, in order to provide decision-making information for traffic commanders. During holidays, Hangzhou “City Brain” collects mobile phone signaling data to conduct real-time flow statistics on key areas of West Lake and deliver the statistics to mobile phone users, which can improve the safety warning mechanism of the scenic spot.

#### Context Awareness.

The application of “City Brain” has improved the quality and efficiency of tourism emergency rescue and tourism services. The “One-click escort” system of “City Brain” can automatically control signal lights to clear the queues in advance through video and real-time information of vehicle trajectory. In this way, it opens up a “green channel” for rescue traffic. The government also lays more emphasis on some “pain spots” of tourism governance. Based on data analysis results, the authorities can provide services such as “find vacancies within 10 s”, “enter the scenic spots within 20 s”, and “annual card for cultural tourism in the Yangtze River Delta” now. Taking the “enter the scenic spots within 20 s” as an example, tourists go to the attraction and open Alipay, scan the payment code and enter the park directly, reducing time spent in queue for ticket purchase.

#### Task Types.

*Numbers.* The “City Brain” cultural and tourism system focuses on the construction of Hangzhou cultural and tourism big data decision-making system. Firstly, build a unified data standard, and a data sharing mechanism to promote the integration of the “city-county-street” three-level data platform. Secondly, rely on the government affairs data sharing and the exchange platform, promote the integration of travel-related data from public security, transportation, urban management and other departments to the “City Brain” cultural tourism system to provide first-hand data support for travel tasks. Finally, in order to accelerate the construction of a tourism data network for vertical and horizontal communication, actively guide tourism companies and other market entities to participate in cultural tourism data production and results sharing.

#### Non-numeric Data.

Hangzhou “City Brain” also analyzed sentiment tendencies on text data such as tourist reviews. For example, the system conducted data mining on hotel reviews from six categories: health, location, service, price, facilities, price, and organized the overall ranking of Hangzhou hotel praise rate and the favorable rate ranking of each individual item.

#### Forms of Artificial Intelligence.

*Digital form.* WeChat applet, APP and other methods have innovated the service process. The WeChat applet of “Hangzhou Digital Tourism Line” relies on various data such as tourist trajectories, railway reservations, and bus operations to plan dedicated lines scientifically and schedule shift times dynamically. At present, 35 special lines have been opened, serving more than 1.2 million tourists. In response to the difficulty of accommodation during holidays, the Hangzhou government has launched a WeChat applet called “Find vacancies”. The front-end vacancy display platform displays the hotel vacancy status; the back-end inventory management system docks with platforms such as PMS and OTA. Tourists can find suitable nearby vacancies based on real-time positioning and price preferences through the applet.

#### Robot Form.

In order to solve the problem of cumbersome check-in and check-out procedures for tourists, the Hangzhou government started from the front desk of the hotel and launched a “hotel check-in in 30 s” service. Tourists can check in and check out quickly by themselves with only three steps: scan and compare ID card, find order and confirm check-in, make room card. Both the check-in experience of tourists and the efficiency of service are improved. At present, “hotel check-in in 30 s” has been connected to 246 hotels, serving more than 284,000 tourists.

### The Impact of Artificial Intelligence on Tourism Enterprises

#### Level of Intelligence.

*Task automation.* Automation does not simply mean replacing manpower with machines, but integrating machines into an autonomous system that can complete a process without manpower assistance [28]. Many tourism suppliers have begun to use artificial intelligence technology in their operations: (1) Automation of customized travel: the company “Mioji Travel” has developed an interface. Users input their destinations, number of travelers and travel preferences. Then they can get a “tailor-made” travel plan in a few seconds. Its founder and CEO Zhang Fan said: *“The process of customized tours should be automated. ‘Mioji Travel’ hopes to automate customized tours through the route planning supported by artificial intelligence.”* (2) Intelligent customer service system: Chinese OTA platforms represented by “Ctrip” and “Qunr” have set up customer service robot systems, which are responsible for answering basic customer service questions.

#### Context Awareness.

Shenzhen timekettle.Co., Ltd. Has developed the world’s first real-time translation headset WT2, which realizes the effect that two people in a conversation do not need to repeatedly pass the device, and do not require the person receiving the conversation to download the program. Its co-founder Qin Zi’ang said, *“When passengers need to ask for flight information or directions, they can talk with foreigners as long as they wear the headset.”* In travel activities such as shopping or dining, this kind of communication will make travel more interesting.

#### Task Types.

*Numbers.* The big data group “Vpon” accurately locates passport holders and high-spending groups by analyzing more than 600 million mobile devices in China. “Vpon” uses AI to research various algorithms to understand the data, and then classify different types of passengers. Since purchase transactions may occur at different stages of travel, people use AI to analyze consumers’ preference during a trip based on the historical data, predict the behavior of passengers, and finally locate advertisements accurately.

#### Non-numeric Data.

The text of customer reviews often contains words of emotional value, such as “thank you”, “sorry”, and “not satisfied”. Some Chinese companies use robots which can detect how many words containing negative emotions the customer has used to collect information about preferences, satisfaction through customer posts on social media platforms.

#### Forms of Artificial Intelligence.

*Digital form.* In July 2019, the “In-depth enjoyment of the Forbidden City tour” applet launched by the Forbidden City was upgraded. The AI tour guide “Mr. Fu” became the biggest highlight. As a cabinet bachelor, “Mr. Fu” can not only chat with tourists, but also recommend personalized tour routes and explain the cultural relics of scenic spots. Especially in this epidemic, a series of “vertical tourism” products represented by the Forbidden City applet played an important role in promoting the recovery of the tourism industry. People can enjoy the beautiful scenery at home.

#### Robot Form.

With technical support, the robot continuously adapts and evaluates application scenarios. In 2018, Alibaba’s first unmanned hotel “Fly Zoo Hotel” landed in Hangzhou. Although full-robot hotels like this may still be rare today, hotels around the world have implemented intelligent automation in some customer-oriented operations, such as automatic check-in, virtual personal assistants, and meal delivery robots.

### The Impact of Artificial Intelligence on Tourist Experience

#### Level of Intelligence.

*Task automation.* In the expectation stage before a trip, there are two main difficulties for tourists in decision-making: First, tourism data is large in volume and diverse in types. The decision-making process consumes energy and requires great ability. Second, the travel process is continuous, so it is difficult to achieve the best time, distance and price at the same time. To solve these problems, the online travel website “Qyer” designed an itinerary planning tool called “Itinerary Assistant”. Tourists input their own needs, and directly perform one-key intelligent optimization to obtain the most reasonable arrangement. In this way, tourists have an increased sense of expectation towards the destination.

#### Context Awareness.

Image recognition, voice recognition, and face recognition technology is widely used in museum tourism in the stage of on-site experience. This article takes the Hunan Provincial Museum, and sorted out 100 key comments with a total of 3507 words. The keywords “technology”, “experience”, “interaction”, and “explanation” appeared 51 times, 21 times, 17 times, and 13 times respectively. *“Admiring modern technology and ancient technology, I often wonder whether the humans who created modern electronic intelligent technology are smarter, or those who created ancient extinctions are smarter. Traveling in the history and imagining the future, this is the surprise and fun brought by cultural relics.”* It can be seen that context awareness technology makes tourists deeply integrate into the scene, breaking their stereotype of the museum. They no longer stay in the sensory aesthetic stage of “skimming the surface”, but develop to a stage of both appreciating the scenery and reflecting on themselves.

#### Task Types.

*Numbers.* The entire process of tourism decision-making, experience, and recall constitutes a closed loop. Before the journey, tourists are usually not clear about their travel preferences, but after in-depth analysis of their previous travel data, they can more clearly recognize their habits and make better decisions.

#### Non-numeric Data.

Still take “Qyer” as an example, it has formed a “Qyer Community” that attracts a large number of experienced tourists. User-produced content (UGC) is its greatest feature. People discover some niche attractions through vivid language and photos. After a trip, they are also willing to share what they have seen and heard. A social community gives tourists a more personalized and memorable experience. Meanwhile, artificial intelligence analyzes the non-numeric data and gains insight into consumer behavior.

#### Forms of Artificial Intelligence.

*Digital form.* In the on-site experience stage, tourists obtain personalized support services through digital products. In Hunan Provincial Museum, tourists can directly scan the QR code on WeChat to get the voice explanation for free, which provides tourists with opportunities to observe cultural relics more closely and experience culture more deeply.

#### Robot Form.

A tourist who has been to the Hunan Provincial Museum commented, *“The kindergarten children are not very interested in the museum, but are attracted by the Xiaodu robot at the entrance.”* For tourists, robots in travel scenes can not only provide more detailed and convenient services, but robots themselves are also an attraction.

## Discussion and the Agenda for Future Research

### Discussion

#### Artificial Intelligence Technology Promotes the Transformation of Tourism Destination Government Management Concepts and Service Models.

In the field of tourism, research similar to artificial intelligence is found in “wisdom tourism” (originating from “smart cities”), and has become an independent research field with the development of “smart cities” [29]. Gretzel et al. [30] decomposed “wisdom tourism” into three main parts: smart experience, smart business ecosystem, and smart destination. It is not difficult to see that “wisdom tourism” is a new field formed by the emerging information communication and artificial intelligence, with a multi-level complex structure. However, the wisdom tourism at the tourism destination level is often led by the government, participated by enterprises and benefited from various aspects. While reforming the tourism industry chain, it also drives the service upgrading of related industries, with dual attributes of commercial service and public service.

#### The Challenge of Technological Progress and the Demand for Experience to Tourism Enterprises.

We can see the impact of different abilities of artificial intelligence on tourism companies in different dimensions in the following aspects: (1) Economic benefits: the use of artificial intelligence robots may bring economic development and various other benefits. Artificial intelligence systems or robots can work 24 h a day. Its low error rate saves costs for enterprises. (2) Internal and external customer service: the impact of this aspect mainly includes two parts: one is forecasting customer needs, conduct customer portraits based on customer past data, and make recommendations based on customer preferences, the other is simplifying repetitive tasks for employees so they can focus on more meaningful things. (3) Tourist satisfaction: a lot of technical research is to improve tourist experience, thereby improving customer satisfaction. (4) Artificial intelligence technology requires enterprises to innovate business models. Companies can understand the preferences of tourists through the analysis of historical data, and even “tailor-made” travel experiences for them.

### The Agenda for Future Research

#### For Destination Management Organization.

How does the tourism destination management structure under the influence of AI technology move from fragmentation to synergy? How will the highly circulated data sharing mode and disintermediated service organization boundaries that accompany the emergence of AI technology help improve the existing power structure? The inefficiency of the market-oriented supply of tourism public services is basically due to the fact that it is difficult to reconcile the contradictions and conflicts between the profit-seeking nature of marketization and the public nature of public services. Promoted by AI technology, how to make the marketized supply of tourism public services move from concealment to transparency, and finally realize the effective supply of tourism public services is worthy of further exploration. How to realize the socialized supply of tourism public services from amateur to professional? It is also a new topic for the development of tourism public services under the background of AI technology to promote the standardization and professionalism of tourism service socialization.

#### For Tourist Enterprises and Tourists.

AI transforms the traditional seller’s market to a buyer’s market. The complex needs of tourists are met, but at the same time privacy issues are encountered. Intelligent systems capture information about tourists on platforms, such as their location. The entire analysis process is a “black box” operation, tourists do not know who uses their information and what channels it is used for.

#### The Interactive Relationship Between Tourists and Virtual Humans.

A kind, patient, and knowledgeable virtual tour guide makes tourists more likely to have a desire to communicate. This kind of interactivity changes the mindset of users. In the future, it is necessary to focus on the social interaction between tourists and virtual people, and to deeply explore its social value and the value of shaping IP.

#### Tourists and the Morality of Robots.

When talking about the erosion and threat of artificial intelligence to people, some researchers will use the term “human-centric” to describe the loss of intrinsic value. Intrinsic value does not serve any purpose, so its influence on moral judgments is crucial. Although the current level of technology is still at the stage of weak artificial intelligence, and only stays at simple technical support, it is still necessary to maintain a rational attitude and make preparations for research on robot ethics related systems.

All in all, this research focuses more on the analysis and summary of the overall research framework, and lacks a quantitative analysis of each case. In the future, we will conduct in-depth analysis on the key cases mentioned.
